# Cell-specific mechanisms of TMEM16A Ca^2+^-activated chloride channel in cancer

**DOI:** 10.1186/s12943-017-0720-x

**Published:** 2017-09-11

**Authors:** Hui Wang, Liang Zou, Ke Ma, Jiankun Yu, Huizhe Wu, Minjie Wei, Qinghuan Xiao

**Affiliations:** 1 Department of Ion Channel Pharmacology, School of Pharmacy, China Medical University, No.77 Puhe Road, Shenyang North New Area, Shenyang, 110122 China; 2 Department of Anesthesiology, National Cancer Center/Cancer Hospital, Chinese Academy of Medical Sciences and Peking Union Medical College, Beijing, 100021 China; 3 Department of Pharmacology, School of Pharmacy, China Medical University, Shenyang, 110122 China

**Keywords:** TMEM16A, Anoctamin 1, Ca^2+^-activated chloride channel, Tumorigenesis, Signaling, Biomarker

## Abstract

TMEM16A (known as anoctamin 1) Ca^2+^-activated chloride channel is overexpressed in many tumors. TMEM16A overexpression can be caused by gene amplification in many tumors harboring 11q13 amplification. TMEM16A expression is also controlled in many cancer cells via transcriptional regulation, epigenetic regulation and microRNAs. In addition, TMEM16A activates different signaling pathways in different cancers, e.g. the EGFR and CAMKII signaling in breast cancer, the p38 and ERK1/2 signaling in hepatoma, the Ras-Raf-MEK-ERK1/2 signaling in head and neck squamous cell carcinoma and bladder cancer, and the NFκB signaling in glioma. Furthermore, TMEM16A overexpression has been reported to promote, inhibit, or produce no effects on cell proliferation and migration in different cancer cells. Since TMEM16A exerts different roles in different cancer cells via activation of distinct signaling pathways, we try to develop the idea that TMEM16A regulates cancer cell proliferation and migration in a cell-dependent mechanism. The cell-specific role of TMEM16A may depend on the cellular environment that is predetermined by TMEM16A overexpression mechanisms specific for a particular cancer type. TMEM16A may exert its cell-specific role via its associated protein networks, phosphorylation by different kinases, and involvement of different signaling pathways. In addition, we discuss the role of TMEM16A channel activity in cancer, and its clinical use as a prognostic and predictive marker in different cancers. This review highlights the cell-type specific mechanisms of TMEM16A in cancer, and envisions the promising use of TMEM16A inhibitors as a potential treatment for TMEM16A-overexpressing cancers.

## Background

TMEM16A (also known as anoctamin 1) was identified as a Ca^2+^-activated chloride channel (CaCC) in 2008 [1–3], and is the first member of the ten-member family of “Transmembrane protein 16” (abbreviated as TMEM16). Besides TMEM16A, TMEM16B and TMEM16F have been found to function as CaCCs [4–6]. TMEM16F can also function as a Ca^2+^-dependent phospholipid scramblase [7, 8], and Ca^2+^-activated nonselective cation channel [9]. However, controversies exist among other members of the TMEM16 family regarding whether they are CaCCs or Ca^2+^-dependent lipid scramblases [7, 10]. The Ca^2+^-dependent lipid scrambling function of TMEM16 family members have been implicated in the regulation of membrane trafficking, the release of extracellular vesicle, and the modulation of cell-cell communication [11].

As a CaCC, TMEM16A is activated by intracellular Ca^2+^, and the current is characterized by voltage-dependent activation, strong outward rectification, and a deactivating tail current on depolarization at low intracellular Ca^2+^ concentrations, and voltage-independent activation and linear current-voltage relationship at high [Ca^2+^]_i_ [12]. Based on the crystal structure of a TMEM16 family member from the fungus Nectria haematococcaten (nhTMEM16), a conserved Ca^2+^-binding site located within the membrane has been identified [13]. Ca^2+^-dependent properties of TMEM16A such as rectification, activation and deactivation kinetics, and rundown can be well explained by the presence of Ca^2+^ binding site within the membrane [14]. However, because nhTMEM16 is a scramblase, not an ion channel, the location of the pore in the TMEM16A channel remains unclear. A “double-barrel” pore architecture of TMEM16A channel has been recently proposed [15, 16].

TMEM16A is widely expressed in many cells including secretory epithelia [1, 17–19], airway and vascular smooth muscle cells [18, 20–22], vascular endothelium [23, 24], interstitial cells of Cajal [25, 26], and nociceptive neurons [27–29]. TMEM16A regulates many cellular functions, such as fluid secretion in secretory epithelia, smooth muscle contraction, gut mobility, cell volume regulation, apoptosis, and pain (reviewed in [30–33]). In addition, TMEM16A dysfunction contributes to many diseases such as cancer, hypertension, gastrointestinal motility disorders, and cystic fibrosis [31, 34–36]. Recently, growing evidence has shown that TMEM16A is overexpressed in many tumors (Table 1). However, conflicting results exist regarding the role of TMEM16A in cell proliferation and migration in cancer cells. In addition, it remains unclear how TMEM16A overexpression contributes to tumorigenesis.

**Table 1:** TMEM16A expression and function in cancers

Author/year	Cancer type	Cancer cells					Human samples	
		Cell lines	Overexpression mechanism	Signaling pathways	Proliferation	Migration	Expression	Clinical outcome of overexpression
Britschgi et al./2013 [42]	Breast cancer	ZR75–1, HCC1954, MDA-MB-415	11q13 amplification	EGFR CAMKII	+	NR	High expression due to gene amplification	Poor survival
Wu et al./2015 [43]	Breast cancer	NR	NR	NR	NR	NR	High expression	Good prognosis in PR^+^ or HER2^−^ breast cancer patients following tamoxifen treatment
Wu et al./2017 [95]	Breast cancer	MCF-7, MDA-MB-435S	NR	NR	+ (MCF-7) - (MDA-MB-435S)	NR	High expression	Good prognosis in PR^+^ or HER2^−^ breast cancer patients the low expression of Ki67
Ubby et al./2013 [94]	Breast cancer	HEK293 cells transfected with TMEM16A	NR	NR	No effect	No effect	mRNA isoforms similar to normal tissue	NR
Duvvuri et al./2012 [44]	HNSCC	UM-SCC1	11q13 amplification	Ras-Raf-Mek-ERK1/2	+	NR	High expression	Poor survival
Ruiz et al./2012 [45]	HNSCC	BHY	11q13 amplification	NR	No effect	+	High expression due to gene amplification	Poor survival
Ayoub et al./2010 [46]	HNSCC	HEp-2 SCC-25	NR	NR	No effect	+	High expression due to gene amplification	Distal metastasis
Shiwarski et al./2014 [74]	HNSCC	UM-SCC1	Low expression of TMEM16A via promoter hypermethylation	NR	NR	–	High expression in primary tumor and low expression in nodal metastatic tissue	NR
Rodrigo et al./2015 [47]	HNSCC	NR	NR	NR	NR	NR	High expression due to gene amplification	No correlation with clinical parameter; affects patient’s survival depending on tumor’s site.
Dixit et al./2015 [73]	HNSCC	HPV-negative FaDu PE/CE-PJ34	NR	NR	+	NR	High expression in HPV-negative HNSCC via promoter hypomethylation	Decreased survival in HPV-negative HNSCC
Bill et al./2015 [79]	HNSCC	Te11	11q13 amplification	EGFR signaling	+	NR	NR	NR
Wanitchakool et al./2017 [59]	HNSCC Colon cancer	BHY, CAL33 HT29, T84	11q13 amplification Epigenetic regulation by HDAC	NR	+ (BHY, CAL33) - (HT29)	+	NR	NR
Shi et al./2013 [50]	ESCC	KYSE30 KYSE510	11q13 amplification	NR	+	NR	High expression due to gene amplification	Lymph node metastasis and advance clinical stage
Cha et al./ 2015 [71]	Prostate cancer	PC3 LnCap	Transcriptional regulation by testosterone	AKT activation	+	NR	High expression	NR
Matsuba et al./2014 [77]	Prostate cancer Breast cancer	PC-3 LNCaP YMB1	Epigenetic regulation by HDAC	NR	+	NR	NR	NR
Mokutani et al./2016 [49]	Colorectal cancer	DLD-1 HCT116	miR-132 downregulation	NR	NR	NR	High expression due to low expression of miR-132	Poor survival
Sui et al./ 2014 [48]	Colorectal cancer	SW620, HCT116 LS174T	Gene amplification	MAPK (MEK and ERK1/2) signaling	+	+	NR	NR
Cao et al./2017 [55]	Gastric cancer	AGS BGC823	MiR-381 downregulation	TGFβ signaling	NR	+	High expression due to miR-381 downregulation	Metastasis and poor prognosis
Deng et al./2016 [52]	Hepatocellular carcinoma	SMMC-7721	NR	MAPK (p38 and ERK1/2) signaling	+	+	High expression	NR
Liu et al./2014 [56]	Glioma	U87MG U251 SHG44 U118	NR	NFκB	+	+	High expression	NR
Jia et al./2015 [51]	Lung cancer	GLC82 NCI-H520	NR	NR	+	+	High expression	NR
Sauter et al./ 2015 [92]	Pancreatic ductal adenocarcinoma	BxPC-3, AsPC-1, Capan-1	NR	NR	No effect	+	NR	NR
Simon et al./2013 [93]	GIST	GIST-T1 GIST-882	NR	NR	No effect	NR	NR	NR
Liu et al./2015 [54]	Gastric cancer	AGS BGC-823	NR	TGF-β signaling	No effect	+	High expression	Poor prognosis

*NR* not reported, + increased, −, inhibited

In this review, we examine recent findings in the study of TMEM16A in cancer, and focus on the role of TMEM16A in cancer cell proliferation and migration. We summarize the mechanisms of TMEM16A overexpression, the signaling pathways that are activated by TMEM16A, and potential clinical use of TMEM16A as a prognostic and predictive marker in cancer. Since TMEM16A plays different roles in different cancer cells, we try to develop the idea that TMEM16A regulates cancer cell proliferation and migration via a cell-specific mechanism.

### TMEM16A Overexpression in cancer

Before it was identified as a CaCC, TMEM16A had been found to be amplified in oral cancer, head and neck squamous cell carcinoma (HNSCC), gastrointestinal stromal tumor (GIST), breast cancer, and esophageal squamous cell (ESCC) cancer under other names such as FLJ10261, TAOS1 (tumor amplified and overexpressed sequence 1) and DOG1 (discovered on GISTs protein 1) [37–41]. Recently, TMEM16A has been reported to be highly expressed in many human tumors including breast cancer [42, 43], HNSCC [44–47], colorectal cancer (CRC) [48, 49], ESCC [50], lung cancer [51], hepatocellular carcinoma [52], prostate cancer [53], gastric cancer [54, 55], and glioma [56] (Table 1).

**Fig. 1:**
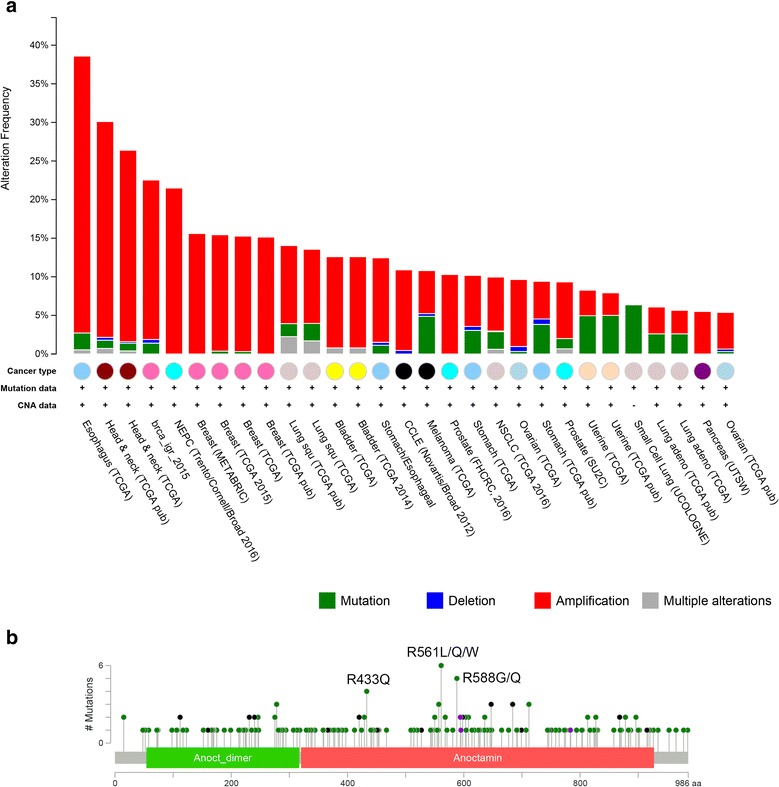
The alterations of the TMEM16A gene in cBioPortal database. **a** TMEM16A gene was examined in 29 studies with >100 human cancer samples and >5% gene alterations. The copy number alteration (CNA) occurs more frequently in cancer. **b** TMEM16A missense mutations identified in cBioPortal database. A total of 165 missense mutations are shown. The most frequent mutations are R561L/Q/W, R433Q, and R588G/Q

TMEM16A is located on chromosome 11q13, which is frequently amplified in many malignant tumors [57, 58]. Several studies have examined the copy number of TMEM16A in many tumors including breast cancer, HNSCC, and ESCC, and found that gene amplification commonly accounts for TMEM16A overexpression in these cancers (Table 1). To further confirm TMEM16A gene amplification in cancers, we performed bioinformatics analysis to detect TMEM16A gene alterations using the cBioPortal database (cBioPortal for Cancer Genomic). TMEM16A gene amplification accounts for the most alterations, and more frequently occurs in HNSCC, ESCC, breast cancer, and lung cancer than in other tumors (Fig. 1a). Interestingly, many tumors have missense mutations and deletions in the TMEM16A gene. A total of 165 missense mutations have been identified in TMEM16A, and the most frequent mutations are R561L/Q/W, R433Q, and R588G/Q (Fig. 1b). However, the role of these mutations has not been investigated in cancer.

Several studies have reported that 11q13 amplification is associated with poor prognosis in patients with malignant tumors [57, 58]. Consistent with the idea, Ruiz et al. found that 11q13 gene amplification correlated with TMEM16A expression in human HNSCC cancer, and TMEM16A overexpression was associated with poor overall survival in HNSCC patients [45]. In addition, Ayoub et al. reported that TMEM16A gene amplification and protein overexpression were associated with distant metastasis in patients with papillomavirus (HPV)-negative HNSCC [46]. Similarly, Bristschgi et al. reported that 11q13 amplification resulted in a higher TMEM16A expression in human breast cancer than in non-11q13-amplified tumors, and TMEM16A gene amplification and protein overexpression correlated with poor prognosis [42]. Shi et al. found that TMEM16A gene amplification and protein overexpression was associated with lymph node metastasis and advanced clinical stage in patients with ESCC [50].

Consistent with the results from the human tumor samples, TMEM16A has been found to be highly expressed in many cell lines with 11q13 amplification, including ZR75–1, HCC1954, and MDA-MB-415 breast cancer cell lines, UM-SCC1, BHY, and Te11 HNSCC cell lines, and FaDu, KYSE30 and KYSE510 ESCC cell lines [42, 44, 50] (Table 1). Knockdown of TMEM16A in cancer cells with 11q13 amplification results in a decrease in cell proliferation and an inhibition in xenograft tumor growth [42, 44, 50, 59]. These studies indicate that TMEM16A is critical for cell proliferation and tumor growth in 11q13-amplified tumors.

Although TMEM16A gene amplification is responsible for TMEM16A overexpression in many tumors, it is clearly not the only mechanism for TMEM16A expression. For example, in breast cancer, 11q13 amplification only occurs in approximately 15% of breast cancer patients, but TMEM16A overexpression occurs in >78% human breast cancer samples [42, 43]. Similarly, TMEM16A overexpression was more pervasive than gene amplification in human gastric cancer samples [54]. In contrast, in HNSCC, TMEM16A gene amplification was more frequently detected than protein expression [45, 47]. Therefore, other mechanisms that regulate TMEM16A expression must exist.

### Multiple regulatory mechanisms of TMEM16A overexpression in cancer

**Fig. 2:**
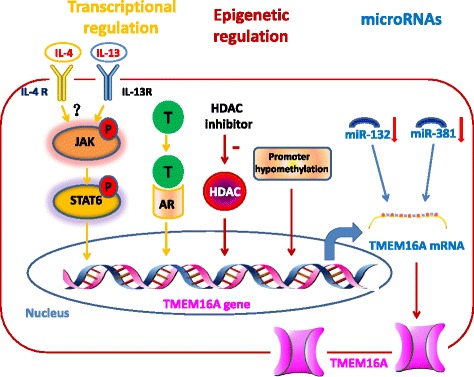
TMEM16A expression is upregulated via transcriptional regulation, epigenetic regulation and microRNAs in cancer. TMEM16A upregulation is induced by IL-4 and IL-13 [64, 65], which bind to their receptors and subsequently activate JAK/STAT6 signaling. STAT6 binds to the TMEM16A promoter and increases the transcription of the TMEM16A gene. Testosterone (T) induces TMEM16A upregulation by binding to the androgen receptor (AR), which subsequently increases the transcription of the TMEM16A gene [71]. Histone deacetylase (HDAC) inhibitors reduce TMEM16A expression in breast and prostate cell lines [77]. Promoter hypomethylation contributed to TMEM16A overexpression in HPV-negative HNSCC [75] and promoter hypermethylation results in decreased TMEM16A expression in metastatic lymph node tissues [74]. miR-132 and miR-381 binds to the 3′ UTR of TMEM16A mRNA, resulting in TMEM16A downregulation [49, 55]. Downregulation of miR-132 and miR-318 contributes to TMEM16A in patients with colorectal cancer [49] and gastric cancer [55]

In non-tumor cells, TMEM16A expression is regulated by many signaling pathways under physiological and pathological conditions. For example, in the airway epithelial cells, IL-4 induces TMEM16A upregulation, which is important for goblet cell differentiation [2, 60]. In human aortic smooth muscle cells, myocardin promotes TMEM16A expression by forming a complex with serum response factor (SRF) on the TMEM16A promoter, and angiotensin II inhibits TMEM16A expression via Kruppel-like factor 5, which competes with SRF to interact with myocardin [61]. In endothelial cells, angiotensin II increases TMEM16A expression [23]. In pulmonary arterial smooth muscle cells, chronic hypoxia increases TMEM16A expression [62]. In human lung epithelial A549 cells, TMEM16A expression is upregulated after lipopolysaccharide treatment [63]. Therefore, it appears that TMEM16A expression is controlled by various molecules and stimuli and the regulatory mechanisms varies in different cells. Here, we summarize the regulatory mechanisms of TMEM16A expression in cancer cells, and TMEM16A expression is controlled via transcriptional regulation, epigenetic regulation, and microRNAs (Fig. 2).

#### Transcriptional regulation

Bioinformatics analyses show that the promoter region of the TMEM16A gene lacks TATAT box sequences, but contains many INRs (initiator elements) and/or TSSs (transcriptional start sites), suggesting that TMEM16A expression can be regulated by diverse transcription factors [64]. The TMEM16A promoter region contains a signal transducer and activator of transcription 6 (STAT6) binding site [64], which mediates TMEM16A upregulation induced by IL-4 and IL-13 [64, 65]. Zhang et al. reported that the expression of TMEM16A and MUC5AC was increased in nasal epithelial cells from patients with chronic rhinosinusitis [66]. IL-13 stimulated MUC5AC expression in human airway and nasal epithelial cells, and this effect was blocked by TMEM16A inhibitors, suggesting that TMEM16A might mediate IL-13-induced mucin secretion [65, 66]. These studies suggest that TMEM16A may play an important role in airway inflammation diseases. It is well known that IL-4 and IL-13 play an important role in cancer development [67–70]. To date, it remains unclear whether TMEM16A can be regulated by IL-4 and IL-13 in cancer cells. Future studies are required to demonstrate whether TMEM16A upregulation by IL-4 and IL-13 is involved in tumorigenesis.

The transcriptional regulation of TMEM16A expression has been demonstrated in testosterone-induced prostate hyperplasia by Cha et al. [71]. They found that the promoter region of the TMEM16A gene contains three putative binding sites for androgen response element (ARE), which mediates testosterone-induced TMEM16A upregulation in prostate epithelial cells. The testosterone-induced TMEM16A upregulation was blocked by small interfering RNAs (siRNAs) against the androgen receptor, which binds to the ARE region and subsequently promotes gene transcription. This study implies that TMEM16A upregulation induced by testosterone may contribute to the progression of prostate cancer.

#### Epigenetic regulation

DNA methylation of the target gene promoter plays an important role in the epigenetic regulation of genes that are essential for tumorigenesis [72, 73]. Promoter hypermethylation can repress gene expression, whereas promoter hypomehtylation can result in active transcription of the gene. TMEM16A promoter contains CpG islands, suggesting that DNA may be involved in the regulation of transcription of the TMEM16A gene [64, 74]. Indeed, Dixit et al. reported that TMEM16A expression was higher in HPV-negative HNSCC than in HPV-positive HNSCC, and promoter hypomethylation contributed to the higher expression of TMEM16A in HPV-negative HNSCC [75]. In addition, Shiwarski et al. found that compared with primary HNSCC tumors, methylation of the TMEM16A promoter region was increased in the metastatic lymph node tissue, thus resulting in decreased TMEM16A expression [74]. Promoter methylation-mediated inhibition of TMEM16A expression is believed to drive HNSCC cells from growth to metastic spread [74].

Histone deacetylase (HDAC) plays an important role in epigenetic regulation of gene expression by deacetylating the lysine residues in the histone, and dysregulation of HDACs has been implicated in the pathogenesis of cancer [76]. Matsuba et al. reported that HDAC inhibitors reduced TMEM16A expression and reduced cancer cell viability in breast and prostate cancer cell lines [77]. Wanitchakool et al. reported that HDAC inhibitors decreased TMEM16A expression and inhibited cell proliferation in HNSCC cells [59]. These studies further suggest that HDAC inhibitors may inhibited cell proliferation via downregulation of TMEM16A. However, the molecular mechanisms underlying the epigenetic regulation of TMEM16A transcription by HDAC have not been elucidated yet.

#### MicroRNAs

MicroRNAs are small, noncoding RNA molecules of ~22 nucleotides that inhibit gene expression by targeting the 3′ UTR of the target mRNAs. MicroRNAs regulate cell proliferation, apoptosis, angiogenesis and invasion, and contribute to tumorigenic processes in human cancers [78]. Recently, Mokutani et al. found that the 3′ UTR of TMEM16A mRNA contained a complementary site for miR-132, and the luciferase reporter assay showed that TMEM16A was the direct target of miR-132 [49]. In addition, TMEM16A overexpression was inversely associated with downregulation of miR-132 in human CRC, and correlated with poor clinical outcomes in patients with CRC [49]. Similarly, Cao et al. found that TMEM16A is the direct target of miR-381, and downregulation of miR-381 was inversely correlated with TMEM16A expression in human gastric cancer tissues [55]. These findings suggest that downregulation of microRNAs may contribute to TMEM16A overexpression in human cancers.

### The signaling pathways activated by TMEM16A in cancer

**Fig. 3:**
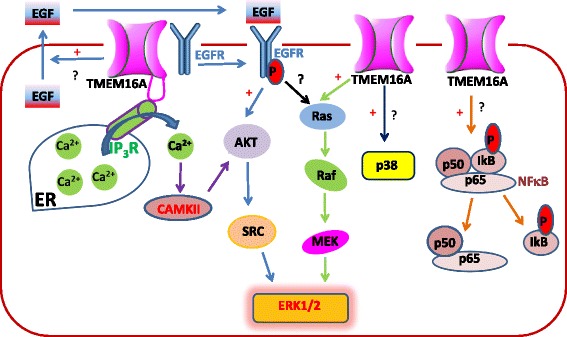
The signaling pathways that are activated by TMEM16A in cancer. TMEM16A directly interacts with EGFR [81], and promotes EGFR phosphorylation, which activates the AKT/SRC/ERK1/2 signaling [42]. In addition, TMEM16A increases autocrine secretion of EGF in breast cancer cells [42]. TMEM16A directly interacts with IP_3_R, and increased Ca^2+^ release from the ER [85]. TMEM16A activates CaMKII by increasing intracellular Ca^2+^ concentrations, and CaMKII subsequently activates the AKT/SRC/ERK1/2 signaling [42]. TMEM16A also activates the Ras-Raf-Mek-ERK1/2 signaling pathway in UM-SCC1 HNSCC cells and T24 bladder cells [44]. In SMMC-7721 human hepatoma cells, TMEM16A activates the p38 signaling pathway [52]. TMEM16A activates the NFκB signaling pathway and promotes the gene transcription in glioma cells [56]. +, activates the signaling pathway.?, the mechanisms of how TMEM16A activates the signaling pathway are unknown

As a CaCC, TMME16A overexpression can result in increased channel function, and opening of TMEM16A chloride channel can lead to changes in intracellular Cl^−^ concentration ([Cl^−^]_i_) and membrane potential. This change in [Cl^−^]_i_ and membrane potential may activate many signaling pathways that are involved in cancer cell proliferation and migration. In addition, as a membrane protein, TMEM16A interacts with several membrane proteins including SNARE proteins that control vesicle trafficking and the ezrin-radixin-moesin (ERM) complex that links membrane proteins with cytoskeleton [79]. It is possible that TMEM16A activates many signaling pathways via its interactome. Here, we summarize the signaling pathways that are activated by TMEM16A in cancer. TMEM16A activates many signaling pathways that participate in cell proliferation, migration, and invasion (Table 1, and Fig. 3).

#### Epidermal growth factor receptor (EGFR) signaling

EGFR is a tyrosine kinase receptor that is overexpressed in many tumors such as HNSCC and breast cancer, and contributes to tumorigenesis [80]. Bill et al. reported that TMEM16A promoted EGFR phosphorylation, and increased the expression in a posttranslational and degradation-independent mechanism in HNSCC cells [81]. In addition, TMEM16A formed a complex with EGFR, and the complex regulated cancer proliferation in HNSCC cells [81]. Activation of EGFR signaling by TMEM16A is further demonstrated by Britschgi et al. showing that TMEM16A knockdown reduced EGFR phosphorylation and subsequently inhibited AKT, SRC, and ERK activation in breast cancer cell lines [42]. Furthermore, they demonstrated that TMEM16A knockdown reduced the autocrine secretion of EGFR ligands, EGF and TGF-α in breast cancer cells, suggesting that TMEM16A can activate EGFR signaling by increasing autocrine secretion of EGFR-ligands [42]. Therefore, TMEM16A activates the EGFR signaling pathway by increasing EGFR expression, phosphorylation, and autocrine EGFR-ligand secretion (Fig. 3).

#### Ca^2+^/Calmodulin-dependent protein kinase II (CAMKII) signaling

Britschgi et al. also found that EGFR inhibition only partially reduced TMEM16A overexpression-induced cell viability, and EGFR activation partially reversed the inhibitory effect of TMEM16A inhibitors on cell viability in breast cancer cells, suggesting that TMEM16A activates additional signaling pathways that are involved in cell viability [42]. Furthermore, they found that TMEM16A overexpression increased calcium/CAMKII phosphorylation, indicating that TMEM16A overexpression activates calcium-dependent CAMKII signaling. It has been reported that TMEM16A is located in the lipid raft of the plasma membrane in nociceptive neurons, where it is in close proximity to IP_3_R [82], and is believed to play a role in modulating intracellular Ca^2+^ levels [83]. In addition, TMEM16A inhibition has been found to reduce intracellular Ca^2+^ flux from both the plasma membrane and sarcoplasmic reticulum in airway smooth muscle [84]. Recently, Cabrita et al. found that TMEM16A directly interacted with the IP_3_R, and increased compartmentalized Ca^2+^ release from the ER store induced by ATP in HeLa cells [85]. TMEM16A inhibitors reduced ATP-induced increase in [Ca^2+^]_i_ [85], suggesting that Cl^−^ transport through TMEM16A channels may be important for Ca^2+^ release from the Ca^2+^ store in cancer cells. In addition, TMEM16A did not interacted with ORAI, and TMEM16A activation was not affected by ORAI inhibitors [85], suggesting that TMEM16A may not regulate ORAI-mediated Ca^2+^ entry in cancer cells.

#### Mitogen-activated protein kinase (MAPK) signaling

The MAPK signaling pathways regulate many cellular processes such as proliferation, apoptosis, migration, differentiation, and growth, and play an important role in the development and progression of cancer [86]. Duvvuri et al. found that TMEM16A overexpression activated the Ras-Raf-MEK-ERK1/2 signaling pathway in UM-SCC1 HNSCC cells and T24 bladder cells, and ERK1/2 inhibition reduced TMEM16A-induced cell growth [44]. However, TMEM16A overexpression did not induced AKT and ERK5 phosphorylation, suggesting that TMEM16A specifically activates the ERK1/2 signaling pathway [44]. Similarly, Sui et al. reported that TMEM16A knockdown decreased the phosphorylation of MEK and ERK1/2 in human colorectal carcinoma cell lines [48]. In addition, in ZR75–1 and HCC1954 breast cancer cell lines, the ERK1/2 signaling pathway is activated by TMEM16A overexpression via EGFR and CaMKII [42]. In SMMC-7721 human hepatoma cells, TMEM16A knockdown reduced the p38 and ERK1/2 phosphorylation, but not JNK phosphorylation, suggesting that TMEM16A activated the p38 and ERK1/2 signaling pathways [52]. Therefore, it appears that TMEM16A predominantly activates the MAPK/ERK1/2 signaling pathway in cancer cells.

#### Nuclear factor κB (NFκB) signaling

The transcription factor NFκB regulates the transcription of many target genes that are involved in a wide range of cellular processes such as cell proliferation, survival, and migration, and plays an essential role in inflammation and cancer [87]. NFκB is a homo- or hetero-dimer that is inactivated by binding to the inhibitory molecule of the inhibitor of κB (IκB) in the cytoplasm in a quiescent cell. IκB phosphorylation releases NFκB dimmers, which subsequently translocate to the nucleus and activate target genes. Liu et al. reported that TMEM16A overexpression resulted in the accumulation of the NFκB subunit p65 in the nucleus, and promoted the transcription of the target genes that involved in cell proliferation, migration, and invasion in glioma cell lines [56]. However, it remains unclear how TMEM16A activates NFκB signaling. It has been reported that EGFR activation promotes NFκB-dependent transcription in ovarian cancer [88]. Since TMEM16A can form a complex with EGFR and activated the EGFR signaling pathway in HNSCC and breast cancer cells [42, 81], it remains to be determined whether TMEM16A activates NFκB signaling via EGFR. In addition, since other chloride channels such as CLC-3 activate NFκB signaling in endothelial cells by lowing [Cl^−^]_i_ [89], it is possible that TMEM16A may also activate NFκB signaling by decreasing [Cl^−^]_i_.

### Cell-specific role of TMEM16A in cancer cell proliferation and migration

#### Cell-specific role in proliferation

TMEM16A overexpression promotes cell proliferation in various cancers such as breast cancer, HNSCC, CRC, ESCC, glioma, lung cancer, hepatocellular carcinoma, and prostate cancer [42, 44, 48, 50–53, 56] (Table 1), and in non-tumor cells such as renal cyst-forming epithelial cells [90] and interstitial cells of Cajal [91]. However, TMEM16A inhibition by shRNAs or pharmacological inhibitors does not affect cell proliferation in BHY HNSCC cells, GIST cells, pancreatic ductal adenocarcinoma cells and gastric cancer cells [45, 54, 92, 93]. In addition, cell proliferation was not affected by overexpression of various breast cancer-specific TMEM16A isoforms in HEK-293 cells [94]. Furthermore, TMEM16A overexpression inhibits angiotensin II-induced proliferation in vascular smooth muscle cells [20, 61], suggesting an inhibitory effect of TMEM16A on cell proliferation in vascular smooth muscle cells. The cell-specific role of TMEM16A suggests that TMEM16A may regulate cell proliferation in a cell-type dependent manner.

However, the differences in the effect of TMEM16A overexpression on cancer cell proliferation may arise from differences in experimental conditions or techniques among studies. To exclude this possibility, Wanitchakool et al. compared the effect of siRNA-knockdown of TMEM16A on proliferation of HNSCC (BHY and CAL33) cells and colonic epithelial (HT29) cells, and found that TMEM16A knockdown suppressed proliferation in HNSCC cells, but not in HT29 cells [59]. Recently, we found that TMEM16A overexpression promoted cell proliferation in ER-positive, PR-positive, and HER2-negative MCF-7 cells, but inhibited cell proliferation in ER-negative, PR-negative, and HER2-negative MDA-MB-435S cells [95]. Therefore, TMEM16A may regulate cancer cell proliferation via cell-specific mechanisms.

#### Cell specific role in migration

Although TMEM16A overexpression does not affect cell migration in HEK293 cells [94], TMEM16A promotes cell migration and invasion in various tumors such as HNSCC, hepatocellular carcinoma, lung cancer, glioblastoma, gastric cancer, pancreatic ductal adenocarcinoma, glioma, oral squamous cell carcinoma, and CRC [45, 46, 48, 51, 52, 54, 56, 92, 96, 97] (Table 1), as well as in non-tumor cells such as bronchial epithelial cells [98] and Ehrlich Lettre ascites (ELA) cells [99]. Interestingly, Jacobsen et al. found that TMEM16A knockdown resulted in a change in the migrating direction in ELA cells, whereas TMEM16F knockdown reduced the speed of migration [99]. The migration and invasion behavior of cancer cells is critical for tumor metastasis and malignancy [46]. The findings that TMEM16A can promote migration and invasion of cancer cells agree with the reports showing that TMEM16A overexpression is associated with lymph node metastasis of ESCC [50] and increased risk of developing metastases in HNSCC [46], and correlates with poor prognosis in patients with breast cancer [42], gastric cancer [54], and HNSCC [45]. However, it has been reported that TMEM16A overexpression has no effects on cell migration when transfected into HEK293 cells [94]. TMEM16A inhibition reduces cell migration in HNSCC cells [74]. Therefore, TMEM16A may regulate cancer cell proliferation via cell type-dependent mechanisms.

Migration of cancer cells requires the change of cell volume by swelling in the front end and shrinkage at the rear end of migrating cells [100]. An osmotic loss of intracellular water via efflux of Cl^−^ through chloride channels can cause cell shrinkage, and thus favor cell migration [101]. It has been reported that TMEM16A facilitates volume regulation and thus promotes cell migration in HNSCC cells [45]. Although the role of TMEM16A in cell volume regulation may explain how TMEM16A promotes cell migration, it is unclear whether TMEM16A alone can promote cell migration. Ubby et al. reported that TMEM16A overexpression in HEK293 cells produced no effect on cell migration, suggesting that the role of TMEM16A in cancer cell migration may be dependent on the intracellular environment of specific cancer cells.

### Cell-specific mechanisms of TMEM16A in cancer proliferation and migration

As discussed above, TMEM16A overexpression in cancer is regulated via multiple mechanisms and TMEM16A activates distinct signaling pathways in different cancer cells (Table 1). These features imply that although most cancers share the same phenotype of TMEM16A overexpression, each cancer may exhibit its unique mechanisms responsible for TMEM16A overexpression and activation of downstream signaling pathways. This heterogeneity among cancer cells may contribute to the cell-specific role of TMEM16A in cancer cell proliferation and migration, as evidenced by several studies showing that TMEM16A overexpression has been found to increase, decrease and produce no effect on proliferation and migration in different cancer cells (Table 1). Cell-specific factors likely contribute to its cell-specific role in cancer, although the exact factors remain largely unknown.

#### Different protein networks associated with TMEM16A

One of the possible reasons for the cell-specific role is that TMEM16A interacts with proteins that are specifically expressed in a certain type of cells. TMEM16A overexpression in HEK293 cells interacts with a wide range of proteins including ERM proteins, GTPases, Ca^2+^-binding proteins, kinases, and lipid-interacting proteins [79]. However, the TMEM16A-associated proteins in HEK293 cells do not include EGFR, which forms a complex with TMEM16A and mediates TMEM16A-induced proliferation in breast cancer and HNSCC cells [42, 81]. Tumor cell-specific expression of EGFR may explain why TMEM16A overexpression promotes proliferation in many tumors expressing EGFR, but not in HEK293 cells [94]. Recently, many other proteins have been found to be associated with TMEM16A in different cells, including 14–3-3gamma and beta-COP in glioblastoma cells [96, 102], Nox2 in endothelial cells [23], and IP_3_ receptors in HeLa cells [85] and nociceptive sensory neurons [82]. Therefore, the cell-specific roles of TMEM16A may be related with its different protein networks in different cells.

#### Phosphorylation by different kinases

The direct protein-protein interaction between TMEM16A and its associated proteins has been found to be dependent on the TMEM16A phosphorylation. For example, the serine 970 (S970) residue in the C-terminal tail mediates the interaction of TMEM16A with radixin, and is required for the effect of TMEM16A on cell morphology and epithelial-to-mesenchymal transition (EMT) [74]. The threonine 9 (T9) residue is important for the interaction of TMEM16A with 14–3-3gamma, and mediates the effect of 14–3-3gamma on TMEM16A surface expression [96]. Although the kinases that phosphorylate these residues in TMEM16A have not been identified, the phosphorylation-dependent regulation of TMEM16A’s interaction with other proteins suggests that the role of TMEM16A may be dependent on the kinase-related signaling in certain cancer cells. It is well known that protein kinases that regulate cell proliferation are dysregulated in cancer and exhibit cell-specific heterogeneity [103, 104]. Therefore, the cell-specific role of TMEM16A may be the result of kinase heterogeneity in different cancer cells.

Migrating cells requires constantly changes in their cytoskeletal arrangement. TMEM16A has been found to directly interact with scaffolding ERM proteins [79], which link the plasma membrane to the actin cytoskeleton and influence cell morphology of cancer cells [105]. ERM proteins are well known to be involved in cancer cell migration, and promote cancer progression [106]. The direct interaction between TMEM16A and ERM proteins suggests that TMEM16A may promote cancer cell migration via ERM proteins. However, Shiwarski et al. found that TMEM16A inhibition increased cell migration and metastasis in HNSCC cells [74]. The inhibitory effect of TMEM16A in cell migration seems contradictory to the migrating-promoting effect of TMEM16A reported in many other studies [45, 46, 48, 51, 52, 54, 56, 92, 96] (Table 1). Since the S970 residue in TMEM16A that is important for direct interaction between TMEM16A and radixin, and is required for TMEM16A’s effect on cell morphology and EMT [74], the activities of kinases or phosphatases that regulate the S970 phosphorylation may be different in different cancer cells, and thus may explain the different results of TMEM16A in cancer cell migration among different studies.

#### Different signaling pathways

The cell-specific role of TMEM16A has also been demonstrated by Britschgi et al., showing that knockdown of TMEM16A resulted in decreased secretion of EGFR ligands (EGF and TGF-α) in breast cancer cells, but not in HNSCC cells [42]. This finding suggests that TMEM16A may activate different signaling pathways in different cancer cells. Indeed, TMEM16A has been reported to activate EGFR and CAMKII signaling in breast cancer cells [42], p38 and ERK1/2 signaling in hepatoma cells [52], Ras-Raf-MEK-ERK1/2 signaling in UM-SCC1 HNSCC cells and T24 bladder cells [44], and NFκB signaling in glioma cells [56] (Table 1), supporting that TMEM16A activates different signaling pathways in a cell type-dependent way.

### Distinct cellular environments may determine the cell-specific role of TMEM16A in different cancer cells

It is obvious that cancer cells with TMEM16A overexpression due to 11q13 gene amplification exhibit different intrinsic cellular environment from those TMEM16A-overexpressing cells without 11q13 gene amplification, since the 11q13 amplicon contains a variety of genes (*CCND1*, *CTTN*, *FADD*, *FGF19)* that regulate proliferation, apoptosis, and cell cycle [107]. This intrinsic genetic expression of these proteins may contribute to cellular changes that drive cancer cells with 11q13 amplification into a proliferating state, in which TMEM16A is important for promoting cell proliferation. In support of this idea, there are consistent results in the literature showing that TMEM16A knockdown in cancer cells with 11q13 amplification decreases cell proliferation in vitro and slows tumor growths in xenograft animals [42, 44, 50, 59], and TMEM16A gene amplification is associated with poor clinical outcome in patients with HNSCC, ESCC, and breast cancer [42, 45, 46, 50] (Table 1). Thus, 11q13 amplification may cause changes in intrinsic cellular environment that favors proliferation-promoting effect of TMEM16A in cancer cells.

Tumor cells are heterogeneous with subpopulation of cells that express different markers and behaviors. Recently, we found that TMEM16A overexpression promoted cell proliferation in ER-positive, PR-positive, and HER2-negative MCF-7 cells, but inhibited cell proliferation in ER-negative, PR-negative, and HER2-negative MDA-MB-435S cells [95]. It appears that the ER and PR signaling pathways may define the cellular environment, in which TMEM16A overexpression promotes cancer cell proliferation. This implicates that particular hormones such as estrogen and progesterone as extrinsic signals may modulate the cellular environment that favors the proliferation-promoting effect of TMEM16A in the subpopulation of cancer cells with ER^+^/PR^+^ status. In agreement with this idea, Cha et al. reported that testosterone upregulated TMEM16A expression via the androgen receptor, and TMEM16A knockdown inhibited testosterone-induced proliferation in prostate cancer cells [71].

DNA methylation status may also be an important factor that affects the cellular environment for determining the role of TMEM16A in cancer. Dixit et al. found that TMEM16A was overexpressed in HPV-negative HNSCC cells, and TMEM16A overexpression increased cell proliferation in HPV-negative HNSCC cells with hypomethylation of TMEM16A promoter, but not in HPV-positive HNSCC cells with TMEM16A promoter hypermethylation [75]. This finding suggests that DNA hypomethylation status may cause changes in cancer cellular environment that favors proliferation-promoting effect of TMEM16A. In contrast, Shiwarski et al. reported that TMEM16A expression was decreased in metastatic lymph node tissues compared with primary tumors in patients with HNSCC, and epigenetical inhibition of TMEM16A expression via promoter hypermethylation was believed to cause cytoskeletal arrangement, increased cell migration and subsequent metastasis [74]. These findings imply that DNA hypermethylation status may define the cellular environment, in which TMEM16A overexpression promotes cancer cell migration and metastasis.

In summary, TMEM16A overexpression in cancer is caused by multiple mechanisms including 11q13 gene amplification, transcriptional regulation, epigenetic regulation, and miRNAs, which also alter the expression of many other proteins and thus may constitute heterogeneity of TMEM16A-overexpressing cancer cells. This heterogeneity may determine unique cellular environment specific for a particular cancer type, in which TMEM16A exerts its cell-specific role in cell proliferation and migration.

### Role of TMEM16A channel activity in cancer

The role of TMEM16A overexpression in cancer cell proliferation and migration has been demonstrated by knockdown of TMEM16A via RNAi-mediated silencing and/or overexpression of TMEM16A-expressing vectors in various cancer cell lines. However, these methods cannot distinguish whether the TMEM16A-mediated roles in cell proliferation and migration are due to increased protein level of TMEM16A or due to increased channel activity. Several studies investigated the role of TMEM16A in cancer cell proliferation using TMEM16A specific inhibitors such as CaCCinh-A01 and T16inh-A01, which inhibits TMEM16A currents [108, 109], and found that pharmacological inhibition of TMEM16A reduced cell proliferation in TMEM16A-overexpressing cancer cells [42, 71, 109, 110]. These studies support that TMEM16A channel function is critical for proliferation-promoting effect of TMEM16A in cancer cells.

However, caution should be taken in drawing conclusions based on the sole use of channel inhibitors, since binding of an inhibitor to TMEM16A may reduce protein stability of TMEM16A, thus reducing the protein level of TMEM16A. For example, a new TMEM16A inhibitor luteolin potently inhibited TMEM16A channel activity and strongly reduced the protein level of TMEM16A [111]. Similarly, CaCCinh-A01 has been found to reduce cancer cell proliferation by promoting loss of TMEM16A proteins via ER-associated, proteasomal degradation of TMEM16A [112]. Therefore, in addition to channel activity, the effect of a TMEM16A inhibitor on protein expression should be investigated, which is often neglected by most studies. In addition, TMEM16A inhibitors can produce nonselective effects. For example, CaCCinh-A01 has been found to inhibit CFTR chloride channels in adult mouse trachea [113], bestrophin-1 chloride channels stably expressed in CHO [114], and Ca^2+^-activated K^+^ channel (KCa3.1) activity in human red blood cells [115]. CaCCinh-A01 and T16inh-A01 has been found to alter intracellular Ca^2+^ concentrations in pancreatic ductal adenocarcinoma cell lines [92] and in HEK293 cells overexpressing TMEM16A [116]. The non-specific effect of a TMEM16A inhibitor should be considered when studying the role of TMEM16A in cancer.

There is also evidence showing that TMEM16A channel activity is not critical for cancer cell proliferation. For example, Seo et al. reported that luteolin, which inhibited both channel activity and protein expression, reduced cell proliferation of PC-3 cells, whereas the luteolin analogue kaempferol, which inhibits TMEM16A channel activity without affecting the protein level of TMEM16A, exhibited weak inhibition on cell proliferation [111]. In addition, several TMEM16A inhibitors that reduced TMEM16A currents but did not affect the protein level of TMEM16A did not inhibit proliferation in TMEM16A-dependent cells [112]. These studies suggest that the protein level of TMEM16A plays a more important role in TMEM16A-induced cell proliferation than its channel activity.

To exclude the possible effect of TMEM16A protein levels on cell proliferation, some studies have investigated the role of TMEM16A on cancer cell proliferation, by overexpression of TMEM16A mutants with altered channel function. They found that TMEM16A mutations with reduced channel function inhibited cancer cell proliferation induced by wild-type TMEM16A [42, 44]. These studies suggest that TMEM16A channel function is at least partially, required for its proliferation-promoting role. Therefore, it appears that both the channel activity and protein level of TMEM16A are important for TMEM16A-induced cell proliferation in cancer cells.

It remains unclear how an increase in TMEM16A protein level and /or channel activity contributes to the role of TMEM16A in cancer cell proliferation and migration. It is possible that TMEM16A overexpression and/or increased channel activity results in changes in [Cl^−^]_i_, which has been reported to regulate cell proliferation in cancer cells [117, 118]. Intracellular Cl^−^ can function as a second messenger, which regulates a wide range of proteins in many signaling pathways [119]. It has been reported that lowered [Cl^−^]_i_ induced by treatment with low Cl^−^ culture medium activates MAPK (including p38 and JNK) signaling via upregulation of the cyclin-dependent kinase inhibitor (p21) in a p53-independent manner in MKN28 gastric cancer cells, thus leading to cell proliferation inhibition [120]. To date, it remains unclear whether TMEM16A overexpression increases or decreases [Cl^−^]_i_. If it is true that TMEM16A overexpression decreases [Cl^−^]_i,_ possibly via increased efflux of Cl^−^, it is possible that TMEM16A overexpression results in activation of the MAPK signaling pathway, which was observed in UM-SCC1 HNSCC cells, T24 bladder cells, and SMMC-7721 human hepatoma cells [44, 52]. However, instead of the proliferation-inhibiting effect of lowered [Cl^−^]_i_ observed in gastric cancer cells by Ohsawa et al. [120], MAPK activation by TMEM16A promotes cell proliferation in UM-SCC1 HNSCC cells, T24 bladder cells, and SMMC-7721 human hepatoma cells [44, 52]. In addition, TMEM16A overexpression activates different MAPK signaling in different cells, e.g. the Ras-Raf-MEK-ERK1/2 signaling pathway in UM-SCC1 HNSCC cells and T24 bladder cells [44], and the p38 signaling pathway in SMMC-7721 human hepatoma cells [52]. This is hard to be solely explained by changes in [Cl^−^]_i_, and it seems that TMEM16A may also regulate other signaling molecules, which modulate different MAPK signaling pathways in different cancer cells.

### TMEM16A as a prognostic and predictive marker

In the clinic, a prognostic marker can be used to predict clinical outcomes of cancer patients in the absence of therapy. TMEM16A has been found to be overexpressed in cancer samples from patients with breast cancer [42, 43], lung cancer [51], oral squamous cell carcinoma [97], esophageal cancer [121], GIST [122], prostate cancer [53], and gastric cancer [54] (Table 1). TMEM16A overexpression is associated with high degree disease and poor overall survival in patients with breast cancer [42]. Gene amplification and protein overexpression of TMEM16A is associated with poor clinical outcomes in patients with HNSCC [45], especially in patients with HPV-negative HNSCC [75]. Recent meta-analyses of microarray datasets have identified TMEM16A as a poor prognostic marker of HNSCC [123]. In addition, TMEM16A overexpression has been found to be associated with poor overall survival in patients with gastric cancer [54], esophageal cancer [121], and CRC [49]. All these studies have found that TMEM16A overexpression is associated with poor prognosis of cancer patients, suggesting that TMEM16A can be used as a prognostic biomarker for clinical outcomes in cancer patients with TMEM16A overexpression.

Recently, we have found that TMEM16A is overexpressed in human breast cancer samples, and TMEM16A overexpression is associated with good prognosis in PR-positive or HER2-negative breast cancer patients following tamoxifen treatment, especially in those patients with the low expression of Ki67 [43, 95]. These findings suggest that TMEM16A overexpression can be used as a predictive biomarker for tamoxifen benefit in patients with PR-positive or HER2-negative breast cancer. TMEM16A expression in combination of clinical relevant markers ER, PR, HER2, and Ki67 may be useful for predicting clinical outcomes of patients with breast cancer. Furthermore, a recent study by Kulkarni et al. has found that TMEM16A inhibition improves responses to EGFR/HER2-targeted therapy in HNSCC cells [110]. Therefore, it is possible that TMEM16A overexpression may be used to predict therapeutic responses of EGFR/HER2 inhibitors in patients with breast and HNSCC cancers.

Li et al. reported that TMEM16A expression in circulating tumor cells (CTCs) was higher in GIST patients with recurrence than that in patients without recurrence, and TMEM16A expression was associated with poor disease free survival [124]. In addition, TMEM16A expression was decreased in GIST patients, who exhibited good responses to imatinib treatment [124]. This study suggests that TMEM16A expression in CTCs can be used as a prognostic marker for monitoring recurrence, and functions as predictive biomarker for evaluating therapeutic efficacy of imatinib treatment in GIST patients.

## Conclusions

TMEM16A overexpression is found in a wide range of human tumors (Table 1). Gene amplification is a major contributor to TMEM16A overexpression in many cancers (Table 1, Fig. 1). TMEM16A gene alterations vary greatly among different tumors (Fig. 1a), suggesting that abnormal TMEM16A gene regulation and/or protein function may be tumor type-specific. In addition, the expression of TMEM16A is also regulated by many other mechanisms, including transcriptional regulation, epigenetic regulation, and microRNAs (Fig. 2). These multiple regulatory mechanisms of TMEM16A expression suggest that TMEM16A expression can controlled by various signaling molecules and stimuli via transcription factors, HDACs, and microRNAs. However, the signaling pathways that regulate TMEM16A expression have not been well established in cancer.

To date, it is still unclear how TMEM16A overexpression contributes to tumorigenesis, and conflicting results exist in the literature regarding the role of TMEM16A in cell proliferation and migration in cancer cells. TMEM16A can be associated with different protein networks, and activates different signaling pathways in different cancer cells (Table 1), suggesting that the cell-specific mechanisms may be responsible for different roles of TMEM16A in cell proliferation in different cancer cells. However, it remains unknown how TMEM16A activates different signaling pathways in different cells. In addition, it is also puzzling whether the protein levels, channel activities, or both are critical for TMEM16A function in cancer cells. As a chloride channel, TMEM16A opening can result in changes in [Cl^−^]_i_ and/or membrane potentials. It is unclear whether changes in [Cl^−^]_i_ and/or membrane potentials contribute to the role of TMEM16A in cancer. Solving these problems will be important for developing a comprehensive understanding of TMEM16A in cancer.

TMEM16A is activated by an increase in [Ca^2+^]_i_ via Ca^2+^ influx through Ca^2+^-permeable ion channels such as voltage-gated Ca^2+^ channels and TPR channels, and via Ca^2+^ release from the endoplasmic reticulum (ER) following activation of G_q_ protein-coupled receptors [14, 125]. TMEM16A couples to different Ca^2+^-permeable ion channels that are predominantly expressed in different cells. For example, TMEM16A has been found to be activated by Ca^2+^ influx via TRPV1 in mouse dorsal root ganglion neurons [28], TRPV4 in the choroid plexus [126], TRPV6 in epithelial principal cells of the rat epididymis [127], TRPC1 in salivary gland cells [128], TRPC2 in rat thyroid cells [129], TRPC6 in cerebral artery myocytes [130], Cav1.4 at the photoreceptor ribbon synapse [131], Cav1.2 in canine ventricular myocytes [132], and store-operated Ca^2+^ entry in eccrine sweat glands [133]. Therefore, TMEM16A is activated by Ca^2+^ via different Ca^2+^-permeable ion channels in a cell-specific manner. However, it remains unclear whether TMEM16A may couple to different Ca^2+^-permeable ion channels in different cancers. Recently, Cabrita et al. have reported that TMEM16A directly interacts with IP_3_R in HeLa cells, and is activated by Ca^2+^ release from the ER via the IP_3_R, but not via ORAI-mediated Ca^2+^ influx [85]. This finding suggests that TMEM16A may be primarily activated by IP_3_R-mediated Ca^2+^ release from the ER in cancer cells. Jin et al. reported a similar finding in small neurons from dorsal root ganglia, showing activation of TMEM16A by IP_3_R-mediated Ca^2+^ release, but not by Ca^2+^ influx via voltage-gated Ca^2+^ channels [82]. Further studies are required to investigate whether IP_3_R-mediated Ca^2+^ release from the ER represents a general mechanism for TMEM16A activation in cancer.

TMEM16A overexpression can be used as a prognostic and predictive marker for clinical outcomes in cancer patients (Table 1). We have previously found that TMEM16A overexpression is associated with good prognosis in PR-positive or HER2-negative breast cancer patients following tamoxifen treatment [43]. Since tamoxifen inhibits TMEM16A currents [1], the beneficial effect of tamoxifen in breast cancer patients may be associated with its inhibition on TMEM16A channel function. In addition, TMEM16A inhibition by T16A-inhA01 and CaCC-inhA01 has been reported to increase responses to EGFR/HER2-targeted therapy in HNSCC cells [110]. Recently, several other TMEM16A inhibitors has been discovered, including MONNA [134], eugenol [135], dehydroandrographolide [136], 9-Phenanthrol [137], Ani9 [138], idebenone [139], and luteolin [111]. Although some TMEM16A inhibitors have been tested in certain cancer cell lines [111, 136, 139], it remains unclear whether these compounds can effectively inhibit cancer growth in vivo, since pharmacological sensitivity of TMEM16A channels may be affected by cellular environment [10, 140, 141]. Both animal and clinical studies are required to investigate the efficacy of a TMEM16A inhibitor on cancer cell growth and metastasis before it can be used for cancer therapy.

## Abbreviations

[Ca^2+^]_i_: Intracellular Ca^2+^ concentration
[Cl^−^]_i_: Intracellular Cl^−^ concentration
ARE: Androgen response element
CaCC: Ca^2+^-activated chloride channel
CAMKII: Calmodulin-dependent protein kinase II
CRC: Colorectal cancer
CTCs: Circulating tumor cells
DOG1: Discovered on GISTs protein 1
EGFR: Epidermal growth factor receptor
ELA: Ehrlich Lettre ascites
EMT: Epithelial-to-mesenchymal transition
ER: Endoplasmic reticulum
ERM: Ezrin-radixin-moesin
ESCC: Esophageal squamous cell
GIST: Gastrointestinal stromal tumor
HDAC: Histone deacetylase
HNSCC: Head and neck squamous cell carcinoma
HPV: Human papillomavirus
INRs: Initiator elements
IκB: the inhibitor of κB
MAPK: Mitogen-activated protein kinase
NFκB: Nuclear factor κB
S970: Serine 970
siRNAs: Small interfering RNAs
SRF: Serum response factor
STAT6: Signal transducer and activator of transcription 6
T9: Threonine 9
TAOS1: Tumor amplified and overexpressed sequence 1
TMEM16: Transmembrane protein 16
TSSs: Transcriptional start sites
